# Differential Response of the Microbiome of *Pocillopora acuta* to Reciprocal Transplantation Within Singapore

**DOI:** 10.1007/s00248-021-01793-w

**Published:** 2021-06-19

**Authors:** Lindsey K. Deignan, Diane McDougald

**Affiliations:** 1 Singapore Centre for Environmental Life Sciences Engineering, Nanyang Technological University, 60 Nanyang Drive, SBS-01N-27, Singapore, 637551 Singapore; 2 The iThree Institute, University of Technology Sydney, Sydney, NSW 2007 Australia

**Keywords:** Urban reef, Coral resilience, Coral microbiology, Coral microbiome

## Abstract

As corals continue to decline globally, particularly due to climate change, it is vital to understand the extent to which their microbiome may confer an adaptive resilience against environmental stress. Corals that survive on the urban reefs of Singapore are ideal candidates to study the association of scleractinians with their microbiome, which in turn can inform reef conservation and management. In this study, we monitored differences in the microbiome of *Pocillopora acuta* colonies reciprocally transplanted between two reefs, Raffles and Kusu, within the Port of Singapore, where corals face intense anthropogenic impacts. *Pocillopora acuta* had previously been shown to host distinct microbial communities between these two reefs. Amplicon sequencing (16S rRNA) was used to assess the coral microbiomes at 1, 2, 4, and 10 days post-transplantation. Coral microbiomes responded rapidly to transplantation, becoming similar to those of the local corals at the destination reef within one day at Raffles and within two days at Kusu. Elevated nitrate concentrations were detected at Raffles for the duration of the study, potentially influencing the microbiome’s response to transplantation. The persistence of corals within the port of Singapore highlights the ability of corals to adapt to stressful environments. Further, coral resilience appears to coincide with a dynamic microbiome which can undergo shifts in composition without succumbing to dysbiosis.

**Supplementary Information:**

The online version contains supplementary material available at 10.1007/s00248-021-01793-w.

## Introduction

Scleractinian corals are the building blocks of ecologically and economically valuable reef ecosystems, yet corals are experiencing ongoing global declines [1, 2]. Coral survival depends on the maintenance of the coral host with its microbial symbionts, the coral holobiont [3]. Corals harbor photosynthetic endosymbiotic dinoflagellates (*Symbiodiniaceae*), which support coral growth through a well-described relationship [4, 5]. Additionally, corals host a diverse microbial community, or microbiome, that plays essential roles in healthy coral functions, including nutrient cycling and immune responses [3, 6]. However, the specific breakdown in functional roles of the microbiome members is not fully understood. The beneficial properties of the microbiome are likely to be contributed by a relatively small group of conserved core microbiome members and a larger group of transient members [7, 8]. Recently, increased research effort has been devoted to better understanding the potential of the microbiome to enable the coral host to withstand environmental perturbations [9, 10].

While coral microbiomes are generally comprised of species-specific communities [11, 12], environmental perturbations like nutrient or temperature fluxes can induce compositional shifts that vary both spatially [13] and temporally [14–17]. It has been proposed that coral species that maintain stable microbiomes may be more tolerant to environmental stressors [18, 19]. For example, the coral *Porites lobata* maintained a stable microbiome during a bleaching event, which supported some protective functions in the host despite the adverse physiological effects of bleaching [20]. However, Pogoreutz et al. [21] found that the stability of the microbiome of *Pocillopora verrucosa* following nutrient enrichment was unable to prevent bleaching or mortality. More frequently, coral microbiome beta diversity is reported to increase with cumulative environmental stressors [16, 17, 22–24], suggesting that a shift in microbiome diversity is needed to counter the effects of long-term stress exposure. Alternatively, it may suggest that the coral lacks the ability to maintain a stable microbial community following perturbation.

Because the coral microbiome appears to contribute to protection of the host during stress, it has been hypothesized that microbiome manipulation, through the inoculation of the coral with presumptively beneficial bacteria, could represent a strategy to enhance coral resilience [9, 10]. However, the functional role of microbial species in maintaining homeostasis in the coral holobiont and the specificity of the microbiome to the local environment are not fully understood. Singaporean corals have adapted to survive intense anthropogenic impacts [25–28], yet the extent to which the coral microbiome contributes to resilience in Singaporean corals remains unclear.

This study focuses on *Pocillopora acuta*, a particularly resilient coral species found in relatively high abundance on reefs throughout Singapore’s Southern Islands [25, 29]. Previous studies have established that *P. acuta* located at Pulau Satumu (also referred to as Raffles Lighthouse) and Kusu Island, two of Singapore’s Southern Islands separated by approximately 15 km, harbor distinct microbial communities, despite their spatial proximities [13, 30]. The waters around the Southern Islands are thought to be well-mixed, but Kusu is located closer to the Singapore mainland and is therefore potentially more exposed to anthropogenic impacts [31]. In this study, we assessed microbiome differences between reefs by monitoring *P. acuta* microbiome following a reciprocal transplantation, as a means of elucidating the microbiome dynamics of corals adapted to environmentally stressful urban reefs.

## Materials and Methods

### Transplantation Experiment

Six colonies of *P. acuta* separated by at least 5 m were selected at random from both Raffles lighthouse (Raffles; 1°09′39″N, 103°44′26″E) and Kusu Island (Kusu; 1°13′32″N, 103°51′35″E; Fig. S1). From each colony, nine 8–10-cm branches were fragmented (all colonies were large enough such that a maximum of one quarter of the colony was fragmented). Two cable ties were used to secure each fragment base to a PVC frame that was mounted approximately 25 cm above the reef benthos (Fig. S2). Following a 72-h acclimation period, one fragment from each colony was sampled representing a day 0 control; after which, four fragments from each colony were transplanted to their reciprocal reef, either transplanted from Raffles to Kusu (Transplant RK) or from Kusu to Raffles (Transplant KR). The remaining four fragments from each colony remained at their resident reef, Resident Raffles and Resident Kusu. One fragment from each colony was sampled at each reef at days 1, 2, 4, and 10 post-transplantation. All collected fragments were immediately placed in a dryshipper for transport to a -80 °C freezer for storage. Coral tissue was collected from each fragment by blasting with compressed air, avoiding the base of the fragment directly under where the cable ties had been fastened. Additionally, on each sampling day, 1-L seawater samples were collected at each reef within 1 m of the transplant frames and placed on ice for transport to the laboratory for filtration through a 0.2-µl filter. All filters were immediately stored at -80 °C. While in the field, 10 ml of 0.2 µl filtered seawater was collected in an acid cleaned falcon tube and immediately stored in a dryshipper for nutrient analysis on an AA3 AutoAnalyzer at the Asian School of the Environment at Nanyang Technological University. Hobo temperature loggers were deployed on each reef to record daily temperatures for the duration of the experimental period.

### DNA Extraction and Sequencing

DNA extraction from the coral tissue and seawater filters was conducted using the Qiagen DNeasy PowerBiofilm kit, followed by the Zymo Clean-up and Concentrator kit. Extracted DNA was stored at -20 °C. PCR was performed with 10 µl HotStarTaq Plus Master Mix, 1 µl each of 10 µM forward and reverse primers, 4 µl water, 1 µl 100% dimethyl sulfoxide (DMSO), 1 µl of 200 ng/µl bovine serum albumin (BSA), and 2 µl template DNA (5 ng/µl). The 515F and 806R primers were used to amplify the V4 region of the 16S rRNA gene to target the prokaryotic community members of the coral microbiomes [32]. Triplicate PCR reactions were run using the following conditions: an initial denaturation at 95 °C for 5 min, followed by 37 cycles of 94 °C for 30 s, 53 °C for 40 s, and 72 °C for 1 min, and a final extension of 10 min at 72 °C. Triplicate samples were pooled for gel extraction. Three blank controls were subjected to identical DNA extraction, amplification, and clean-up methods. All samples were quantified using a Qubit 2.0 fluorometer and quality checked on an Agilent 2200 TapeStation before transferring to the Singapore Centre for Environmental Life Sciences Engineering, Nanyang Technological University, for library preparation and amplicon sequencing on an Illumina MiSeq platform. Raw sequencing reads were uploaded to the NCBI Sequence Read Archive (SRA) under BioProject PRJNA667314.

Amplicon sequencing data were processed using Dada2 version 1.16 to generate amplicon sequence variants (ASVs) for each sample replicate. Briefly, paired sequence reads were trimmed to 200 for the forward read and 170 for the reverse read and then filtered with an expected error rate of 2. Error learning algorithms were applied to the forward and reverse reads before reads were merged, and chimeric sequences were removed. Contaminating sequence reads based on comparison with blank extractions were removed using the *decontam* package [33]. Taxonomy was assigned to the genus level using the IDTAXA algorithm from the R package DECIPHER v 2.16.1 based on the SILVA SSU r138 database [34]. Sequences identified as mitochondria, chloroplast, or unassigned to a Domain were removed. Due to situational limitations, a portion of the laboratory sample processing was conducted from July to November 2019, and the remaining sample processing was conducted from March to July 2020. As an additional control measure, Dirichlet Multinomial Mixtures (DMM) models of the day 0 control samples and resident colonies from Kusu and Raffles were used to confirm the removal of sample processor bias based on the temporal separation of sample processing using the *DirichletMultinomial* package in R [35]. DMM models were restricted to ASVs with > 0.1% relative abundance in 50% of the samples to target contamination that would be present at an abundance with the potential to bias results, without accounting for the lower abundant ASVs that contribute to the site differences between Raffles and Kusu. The models were run for up to 6 potential Dirichlet mixture components, and a Laplace model was used to test goodness of fit [36]. Initial DMM models performed on the sequencing data grouped the samples into 2 Dirichlet mixture components, or clusters, which matched directly to processing year (Fig. S3). Four ASVs were found to correlate directly to processing year; two *Pseudomonas* spp. were only present in the samples processed in 2019, and two *Ralstonia* spp. were only present in high abundance in the samples processed in 2020. Due to the direct correlation of these ASVs with processing year, they were removed as contaminants. Confirmation of these ASVs as contaminants and not contributing to experimental results was verified with follow-up DMM models, which grouped the coral samples into one Dirichlet mixture component following removal of the 4 ASVs (Fig. S4). Finally, rarefaction curves were used to assess samples which reached their ASV maximum, and five samples were removed for failing to meet asymptotic levels (Fig. S5). The remaining samples were rarefied to 8416 sequence reads per sample to account for variation in sequencing depths for the calculation of diversity metrics [37]. The code used to generate the final dataset is available at https://github.com/LindseyKDeignan/Pocillopora_transplantation.

### Data Analysis

To examine the patterns of coral-associated prokaryotic communities, non-metric multidimensional scaling (nMDS) plots were created using Bray–Curtis similarity matrices of square root transformed data with the *Vegan* package in R v3.4.3. Permutational multivariate analyses of variance (PERMANOVA) were performed using the PERMANOVA + add-on in PRIMER 7 to compare between coral fragment origin and location over time. Analyses of location were used to compare the resident fragments and the fragments transplanted to that location with those on the opposite reef (Resident Raffles and Transplant KR vs. Resident Kusu and Transplant RK). Analyses of origin were performed to compare the resident fragments and the fragments transplanted away from that location to the fragments that originated from the opposite reef (Resident Raffles and Transplant RK vs. Resident Kusu and Transplant KR). Permutational multivariate analysis of dispersion (PERMDISP) was used to test for homogeneity of dispersion among samples within groups. The PERMDISP analysis can also be used as a measure of beta diversity between sample groups [23]. Multiple pairwise comparisons were corrected based on the Benjamini and Yekutieli (B-Y) false discovery rate control [38].

To determine the specific ASVs that contributed to the differences detected by PERMANOVA, multivariate generalized linear models (GLMs) with negative binomial distribution were performed in R v 3.4.3 using the *mvabund* package. Each dataset was subsampled to 500 OTUs for the GLM analysis. These analytical methods were first used to establish a difference in the prokaryotic communities of the coral microbiomes between the two reef sites and then among resident coral microbiomes within each site individually over the 10-day experimental period. The GLMs were also used to compare the resident coral microbiomes to those of the transplanted coral fragments where differences were detected with PERMANOVA.

## Results

Coral fragments remained visually healthy at both sites for the duration of the experiment, with few exceptions. Corals originating from Raffles maintained a slightly lighter coloration, without bleaching, throughout the experiment as compared to fragments originating from Kusu. Four Resident Kusu fragments died. Among the transplanted colonies, 1 Transplant RK and 2 Transplant KR were dead by day 10. Death of a fragment was defined as > 80% tissue loss. Of the surviving fragments, 8 failed to meet sequencing criteria and were excluded from the analysis, resulting in 92 total fragments analyzed (Table S1).

**Fig. 1:**
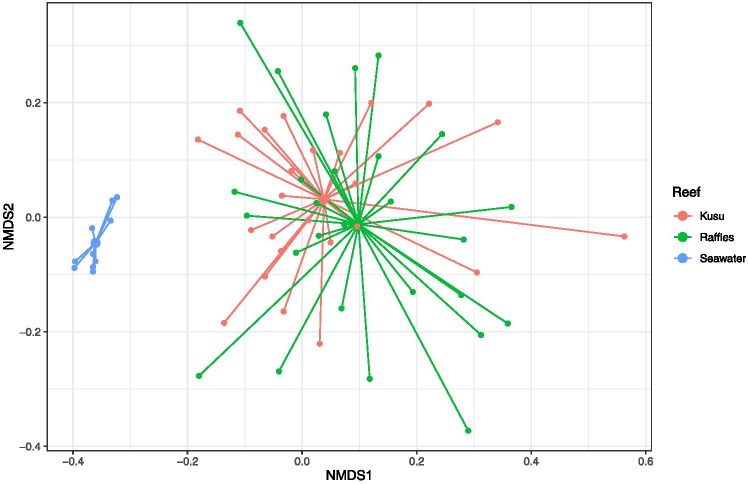
nMDS plot of all Seawater and Resident Raffles and Kusu samples for all sampling time points of the transplantation experiment graphically represented as a spider plot connecting each sample with the group centroid

**Fig. 2:**
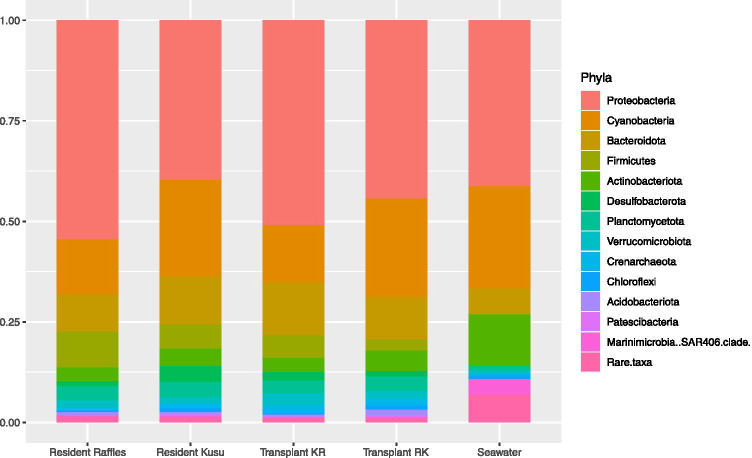
Mean relative abundance of phyla in all resident, transplanted, and seawater samples. The seawater group includes seawater from Raffles and Kusu

**Fig. 3:**
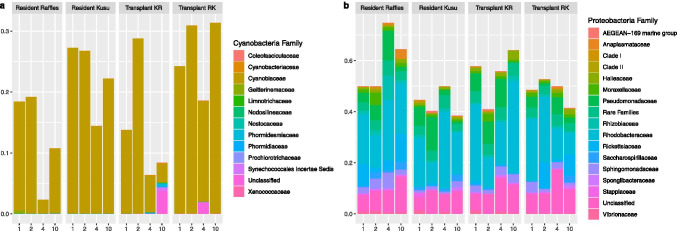
Mean relative abundance at the family level of the two dominant taxa, **a** Cyanobacteria and **b** Proteobacteria, in the coral microbiomes for each sampling day following transplantation

Coral microbiomes of the Resident Raffles samples were significantly different from the Resident Kusu samples (PERMANOVA, pseudo-F = 2.0566, P = 0.001; Fig. 1). Coral samples were distinct from seawater samples (PERMANOVA, pseudo-F = 11.262; P = 0.001), while seawater samples from each reef were not significantly different (PERMANOVA, pseudo-F = 1.5053; P = 0.145). The microbiomes of coral fragments at all time points were dominated by *Proteobacteria*, followed by *Cyanobacteria* (Fig. 2). At Raffles, there was a higher relative abundance of *Proteobacteria* for both the Resident and Transplanted fragments, while at Kusu, the Resident and Transplanted fragments had a relatively higher abundance of *Cyanobacteria*. The *Cyanobacteria* were composed almost exclusively of *Cyanobacteriaceae*, while the *Proteobacteria* were represented by high proportions of *Rhodobacteraceae* and unclassified families (Fig. 3). Alpha diversity metrics, including richness, Chao1, Shannon diversity (H), and Inverse Simpson, were not significantly different between sites or for transplanted fragments (Fig. S6).

Coral microbial communities had limited shared ASVs among samples. When an ASV was present in > 90% of all coral samples, it was considered to be a core member of the microbiome. Based on this definition, there were eight core ASVs, 6 of which belonged to the family *Cyanobiaceae* and 2 to the family *Rhodobacteraceae*. When examining Raffles and Kusu separately, the six core microbial members were present in each reef, except a reduction of one *Cyanobiaceae* at Raffles and an increase of three additional *Cyanobiaceae* at Kusu. If the core microbiome was redefined as ASVs present in > 70% of all coral samples, the number of core microbiome members shared across all samples at both reefs increased to 15, with additional ASVs including one *Flavobacteriaceae*, three *Rhodobacteraceae*, and three *Cyanobiaceae*. For Raffles, the core microbiome contained 16 members, when defining the core microbiome as presence in 70% of samples, and for Kusu, the number of ASVs within the core microbiome increased to 21. At Raffles, the 16 shared ASVs were made up of 8 *Cyanobiaceae*, 6 *Rhodobacteraceae*, 1 *Flavobacteriaceae*, and 1 from class *Actinobacteria*. At Kusu, the 21 shared ASVs were partitioned as 12 *Cyanobiaceae*, 5 *Rhodobacteraceae*, 2 *Flavobacteriaceae*, and 2 from class Actinobacteria.

Two-factor PERMANOVAs detected significant differences based on location (Raffles or Kusu) and time (days 1, 2, 4, 10) for each factor and interaction. Repeated analyses using origin (Raffles or Kusu) and time (days 1, 2, 4, 10) as factors also detected significant differences (Table S2). One day after transplantation, coral fragments remained differentiated by their source reef (origin); i.e., resident colonies remained similar to those that had been transplanted away from the source reef (Table 1). However, by the second day post-transplantation, the transplanted coral fragments were no longer similar to the resident colonies on their reef of origin but were instead more similar to their transplanted location. When assessing the response to transplantation within each reef separately, the microbiome shift happened more quickly for coral fragments transplanted from Kusu to Raffles (Table 1). There was no differentiation detected between the Resident Raffles and Transplant KR samples, but on day 1, there was a difference between the Resident Kusu and Transplant RK samples. When comparing homogeneity, or dispersion, among groups, PERMDISP analysis found increased dispersion for corals associated with Raffles, which led to a significant reduction in dispersion for corals that were transplanted from Raffles to Kusu (Table S3). Dispersion from the centroid was calculated for samples in the following groups: Resident Raffles (58.34 ± 1.03), Resident Kusu (55.17 ± 0.99), Transplant RK (53.12 ± 0.94), and Transplant KR (58.13 ± 0.83).

**Table 1:** Pairwise PERMANOVA comparing the microbiome community structure of the coral fragments at 1, 2, 4, and 10-days post-transplantation, first comparing the fragments based on the reef from which the corals originated (Origin) and secondly comparing the coral fragments based on the reef on which they were located on at the time of sampling (Location). The next comparisons are separated within each respective reef, comparing the Resident Raffles fragments to the Transplant KR fragments (Raffles), followed by a comparison of the Resident Kusu fragments compared to the Transplant RK fragments (Kusu). The test statistic is included, and italics indicate significant differences based on corrected P values

	Origin		Location		Raffles		Kusu	
	t	*P*	t	*P*	t	*P*	t	*P*
Day 1	1.2354	*0.001*	1.0994	0.034	1.1074	0.033	1.2237	*0.004*
Day 2	1.1106	0.091	1.1818	*0.024*	1.0150	0.328	1.1276	0.089
Day 4	1.0184	0.331	1.1891	*0.001*	1.0478	0.112	1.1015	0.086
Day 10	1.1393	0.046	1.3019	*0.001*	1.1789	0.218	0.9991	0.469

**Fig. 4:**
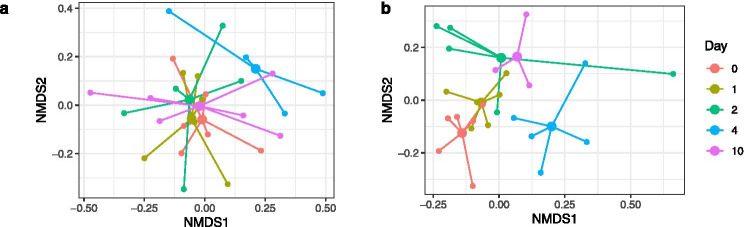
nMDS plot of the **a** Resident Raffles and **b** Resident Kusu samples at each sampling day graphically represented as a spider plot connecting each sample with the daily group centroid. One sample was removed on day 4 at Raffles to increase readability of the nMDS plot (see Fig. S7 for nMDS plot with sample included)

Resident coral microbiomes from each reef also changed during the 10-day experimental period (Table 2). At Raffles, there was a significant shift in the microbial community composition of the day 4 and day 10 resident fragments as compared to days 1 and 2 (Fig. 4a).

These results might be partially explained by the relative abundance of *Cyanobacteria* compared to *Proteobacteria* on day 4 (Fig. 3). For Kusu, the microbiome shift was apparent at day 2 and continued to day 4. However, the day 10 samples were not distinct from the earlier sampling time points (Fig. 4b). There were no significant changes in dispersion within each site during the experiment (Raffles, F = 2.5395, P = 0.144; Kusu, F = 1.8881, P = 0.556); however, dispersion was higher for Resident Raffles and Transplant KR samples, compared with the Resident Kusu or Transplant RK samples (Table S3).

**Table 2:** Pairwise PERMANOVA comparing microbiome community structure of the Resident Raffles and Resident Kusu microbiomes within each reef over time. The test statistic is included, and italics indicate significant differences based on corrected P values

	Raffles		Kusu	
	t	*P*	t	*P*
Day 0, day 1	1.0036	0.436	1.1225	0.052
Day 0, day 2	1.0035	0.428	1.2789	*0.010*
Day 0, day 4	1.2071	*0.003*	1.2935	*0.002*
Day 0, day 10	1.1671	*0.015*	1.2537	0.029
Day 1, day 2	0.9906	0.591	1.1854	*0.006*
Day 1, day 4	1.1439	*0.007*	1.2577	*0.002*
Day 1, day 10	1.1344	*0.012*	1.1118	0.303
Day 2, day 4	1.0933	0.078	1.1725	0.047
Day 2, day 10	1.1081	0.094	1.0781	0.325
Day 4, day 10	1.0986	0.063	1.0960	0.349

There were 27 Bacterial ASVs and 1 Archaeal ASV identified as highly significant (*P* < 0.01) based on the GLM in driving the differences among resident and transplanted corals and occurring with a mean relative abundance of ≥ 0.1% in at least one of the four treatment groups (Table 3). Notably, *Cyanobacteria* was elevated at Kusu as compared to Raffles, and *Cyanobacteria* remained as the dominant taxa in the coral fragments that were transplanted from Kusu to Raffles. Corals from Kusu had elevated abundances of ASVs of *Actinobacteriota*, while those at Raffles had elevated *Firmicutes*, which were each acquired by fragments from the opposite reef following transplantation. Corals from Raffles and Kusu each had specific *Bacteroidota* ASVs that were retained in the transplanted fragments. *Proteobacteria* ASVs were primarily elevated among transplanted Raffles coral fragments; however, there was one ASV (ASV60) that was elevated in Kusu originating corals (Table 3). Two *Verrucomicrobiota*, closely related to the genus *Simkania*, were present in the Resident Kusu and Transplant KR fragments. The one significant Archaea identified by the GLM (Family *Nitrosopumilaceae*) had the lowest abundance in the Resident Raffles coral fragments.

**Table 3:** The percent abundance with standard deviation of the top (> 0.1% of total microbial community) ASVs identified as highly significant (*P* < 0.01) in driving the differences between resident and transplanted corals based on GLM

ASV	Classification	Resident Kusu	Transplant KR	Resident Raffles	Transplant RK
	*Actinobacteriota*				
47	Order PeM15	0.436 ± 0.427	0.049 ± 0.102	0.263 ± 0.307	0.692 ± 0.526
52	Order PeM16	0.448 ± 0.452	0.131 ± 0.213	0.233 ± 0.360	0.647 ± 0.548
159	Class *Actinobacteria*	0.086 ± 0.144	0.013 ± 0.049	0.098 ± 0.275	0.305 ± 0.288
	*Bacteroidota*				
16	* Maritimimonas* sp.	1.447 ± 1.710	1.679 ± 1.845	0.097 ± 0.196	0.018 ± 0.058
18	* Maritimimonas* sp.	1.287 ± 1.401	1.572 ± 1.792	0.129 ± 0.258	0.017 ± 0.077
231	Family *Flavobacteriaceae*	0	0	0.115 ± 0.233	0.163 ± 0.455
254	Family *Flavobacteriaceae*	0	0	0.118 ± 0.254	0.153 ± 0.352
430	Family *Flavobacteriaceae*	0	0	0.186 ± 0.510	0.016 ± 0.066
	*Cyanobacteria*				
3	* Cyanobium* PCC-6307	3.442 ± 2.054	1.228 ± 1.645	1.390 ± 1.384	4.216 ± 3.207
4	* Cyanobium* PCC-6307	3.824 ± 2.559	1.353 ± 1.884	1.493 ± 1.589	3.951 ± 2.735
22	* Synechococcus* CC9902	0.808 ± 0.641	0.341 ± 0.527	0.276 ± 0.421	0.835 ± 0.506
25	* Synechococcus* CC9902	0.871 ± 0.733	0.374 ± 0.488	0.292 ± 0.417	0.777 ± 0.532
	*Firmicutes*				
217	Family *Peptostreptococcaceae*	0	0.235 ± 0.577	0.178 ± 0.418	0.015 ± 0.039
236	* Romboutsia* sp.	0	0.196 ± 0.436	0.213 ± 0.415	0.009 ± 0.040
295	Family *Peptostreptococcaceae*	0	0.201 ± 0.802	0.063 ± 0.137	0
325	Family *Peptostreptococcaceae*	0	0.147 ± 0.629	0.081 ± 0.186	0
	*Proteobacteria*				
23	Family *Rhodobacteraceae*	0.305 ± 0.456	0.078 ± 0.137	0.901 ± 1.575	1.508 ± 3.262
24	Family *Rhodobacteraceae*	0.233 ± 0.355	0.084 ± 0.137	0.798 ± 1.185	1.336 ± 2.450
60	Family *Rhodobacteraceae*	0.502 ± 0.898	0.434 ± 0.571	0.090 ± 0.250	0.054 ± 0.151
81	* Erythrobacter* sp.	0.058 ± 0.133	0.358 ± 0.539	0.244 ± 0.342	0.342 ± 0.360
151	Class *Gammaproteobacteria*	0	0.069 ± 0.303	0.191 ± 0.361	0.243 ± 0.397
152	Class *Gammaproteobacteria*	0.003 ± 0.014	0.100 ± 0.397	0.190 ± 0.309	0.175 ± 0.286
189	Family *Rhodobacteraceae*	0.064 ± 0.147	0	0.164 ± 0.494	0.118 ± 0.253
191	Family *Rhodobacteraceae*	0.056 ± 0.116	0	0.174 ± 0.426	0.104 ± 0.188
230	Family *Halieaceae*	0	0.049 ± 0.143	0.135 ± 0.487	0.066 ± 0.131
	*Verrucomicrobiota*				
165	* Simkania* sp.	0.286 ± 0.724	0.180 ± 0.487	0.003 ± 0.011	0.002 ± 0.008
180	* Simkania* sp.	0.230 ± 0.646	0.241 ± 0.675	0	0
	*Crenarchaeota*				
113	Family *Nitrosopumilaceae*	0.200 ± 0.302	0.150 ± 0.293	0.015 ± 0.049	0.352 ± 0.905

**Fig. 5:**
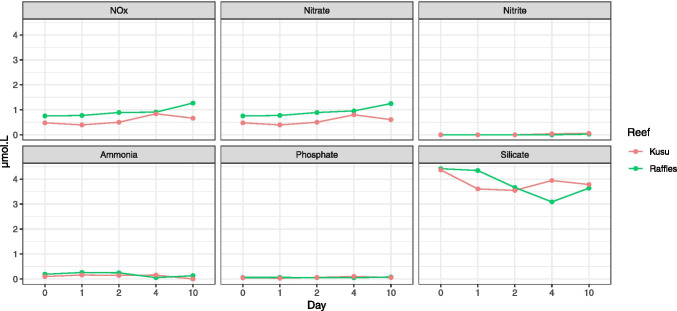
Seawater nutrient concentrations at Raffles and Kusu during the experiment

Seawater nitrite, ammonia, phosphate, and silicate concentrations were not different between Raffles and Kusu during the 10-day period (Table S4). However, nitrate was significantly higher at Raffles (0.9262 ± 0.1994 µmol/L) versus Kusu (0.558 ± 0.1563 µmol/L; Fig. 5). Seawater temperature was not significantly different between reefs (30.27 ± 0.12 °C at Raffles and 30.31 ± 0.18 °C at Kusu; T-test: t = 0.6580, df = 23.013, P value = 0.517).

## Discussion

The coral microbiomes of *P. acuta* examined in this study were dynamic in response to both spatial and temporal changes. Coral microbiomes of both reefs exhibited temporal shifts during the experimental period. Coral microbiomes also responded rapidly, though differentially, to transplantation. Corals that were transplanted from Kusu (Transplant KR) were similar to the Resident Raffles corals within 1 day post-transplantation, while the colonies that were transplanted from Raffles (Transplant RK) were similar to the Resident Kusu colonies within 2 days post-transplantation. Coral samples from Raffles, as well as the Transplant KR fragments, displayed greater dispersion, or increased beta diversity, than fragments transplanted to Kusu, which may have contributed to the different rates of microbiome response to transplantation. Corals originating from Raffles may have taken longer to become similar to the Resident Kusu corals, because the Raffles corals were comprised of more divergent microbial communities. Conversely, the transition in microbiomes of corals that were transplanted from Kusu to Raffles may have happened more quickly, because the samples from Kusu were a less divergent community that became more dispersed.

Corals from both locations had microbiomes dominated by phylum *Proteobacteria*, followed by phylum *Cyanobacteria*. These results are consistent with a previous study, which characterized *P. acuta* from Kusu as having a high abundance of both *Proteobacteria* and *Cyanobacteria* [13]. However, a similarly high abundance of *Cyanobacteria* was not previously observed in *P. acuta* from Raffles. The high abundance of *Cyanobacteria* found in corals from Raffles in this study could be linked to the elevated nitrate concentrations observed there, as *Cyanobacteria* can process nitrate and has been positively correlated with nitrate concentrations [39]. Elevated *Cyanobacteria* abundance has been associated with coral reef ecosystem degradation, and some *Cyanobacteria* can be a precursor to coral disease [40, 41]. However, none of the abundant *Cyanobacteria* found in this study were previously identified as pathogenic to corals. *Cyanobacteria* were also found in the seawater from both Raffles and Kusu, so it is possible that some *Cyanobacteria* identified from the coral samples were surface-associated bacteria and not incorporated into the coral microbiome, as the mucus layer can more readily incorporate environmental bacteria [11, 42]. While samples examined in this study were composed primarily of coral tissue, some mucus was likely included. However, given that *Cyanobacteria* have been previously reported in the microbiome of *P. acuta* tissue from Kusu, at least some *Cyanobacteria* may be persistent associates of the coral microbiome.

Coral transplantation can facilitate the colonization of disease pathogens in corals [43], although there was no apparent increase in potentially pathogenic bacteria in the transplanted fragments. Two *Verrucomicrobiota*, most closely matching the genus *Simkania*, were present in the Resident Kusu fragments and retained by the Transplanted KR fragments; however, the two bacteria were not acquired by corals transplanted from Raffles to Kusu. The genus *Simkania* currently contains one species, *S. negevensis* [44], which has been implicated as a human pathogen [45]. Both strains of *Simkania* spp. had 98% and 97.8% identity, respectively, to *S. negevensis*, suggesting they represent a closely related yet undescribed *Simkania* species. Given the potential association of these bacteria with human infections, it is notable that they only occurred in corals originating from Kusu, the reef located closer to the mainland of Singapore.

In addition to the shift induced by transplantation, we observed natural shifts in the resident coral microbiomes over the 10-day experiment. While there is evidence of coral microbiomes responding quickly to stress [15, 46], there is limited information about the dynamic nature of coral microbiomes from day to day. This shift in microbiome community structure could be a response to fragmentation of the resident colonies, which were fragmented similar to the transplanted samples. However, there was no change in beta diversity of the resident colonies within each reef over time, suggesting that the microbiome shifts reflect natural fluctuations in the coral microbial communities at each site. There was a slight increase in NO_x_ during the study, which could have contributed to the temporal changes in coral microbiomes. In particular, the differences in nitrate concentration detected between the reefs could contribute to site-specific differences, as nitrogen concentrations remained elevated at Raffles compared to Kusu for the duration of the experiment. In contrast, no other water quality parameters measured in this study, including temperature or nutrient concentrations, can fully explain the temporal microbiome changes observed. Future studies should monitor a broader range of environmental parameters.

Of the two reefs compared in this study, Kusu is generally considered to experience greater anthropogenic impacts than Raffles, due to its proximity to the mainland of Singapore (~ 4 km from mainland Singapore versus ~ 13 km, respectively). Previous monitoring reported greater water movement at Raffles as compared to Kusu, with no significant differences observed between sites for sedimentation rate, temperature, or nutrient concentrations [47]. Wainwright et al. [13] attributed differences in coral microbiomes between Raffles and Kusu to the strong directionality of water movement from east to west differentially affecting the windward and leeward sides of Singapore’s Southern Islands, particularly during the Northeast Monsoon [31]. The current study was conducted during the inter-monsoon period with light winds and relatively calm seas, which may explain why the coral microbiomes between the two reefs were not as divergent as in previous studies [13, 30]. The mean seawater temperature of approximately 30 °C recorded at each reef and the nutrient concentrations are consistent with expectation for Singaporean waters [31, 48]. However, the elevated nitrogen concentrations around Raffles may have contributed to the higher beta diversity observed for corals within that site [24, 49], if they represented anomalous nitrogen loading in the system. While only a small fraction of water quality parameters characterizing the two reefs were accounted for in this study, even in urbanized reef communities, environmental variables are not always explanatory of microbiome composition [50].

Corals in Singapore are adapted to an environment of high disturbance, which is often associated with an increased beta diversity of the microbiome [16, 17, 22–24]. In this study, the microbiome of *P. acuta* readily shifted, but no dysbiosis, or shift to disease state, was observed. These findings are consistent with *P. acuta* from the Great Barrier Reef, in which the microbiome changed in composition in response to a thermal stress event [19], although the differences observed on the Great Barrier Reef were not as pronounced as those reported here. The results of this study support the hypothesis that the coral microbiome is highly dynamic, readily shifting in response to changes in the environment, with only a small group of core microbial members. The dynamic microbiome of *P. acuta* in Singapore may be an adaptation to the environmental fluctuations consistent with the intense anthropogenic impacts experienced on Singapore’s urban reefs. Additional studies are needed to elucidate the exact role of individual microbial members on coral health and to determine whether the microbiome variations have a net negative or positive effect on long-term survival. Thus, corals from Singapore represent a model of resilience and therefore understanding how their microbiomes have adapted to the environment may yield clues to their persistence on these heavily impacted reefs.

## Supplementary Information

Below is the link to the electronic supplementary material.

Supplementary file1 (DOCX 909 KB)

## Data Availability

Raw sequencing reads were uploaded to the NCBI Sequence Read Archive (SRA) under BioProject PRJNA667314.
