# Supplementary material for: Differential Response of the Microbiome of Pocillopora acuta to Reciprocal Transplantation Within Singapore
Source: Microb Ecol. 2021 Jun 19;83(3):608–18. doi: 10.1007/s00248-021-01793-w (PMC8979861; doi:10.1007/s00248-021-01793-w)
Supplement: Supplementary file 1 — Supplementary file1 (DOCX 909 KB) [file 248_2021_1793_MOESM1_ESM.docx]

**Differential response of the microbiome of *Pocillopora acuta* to reciprocal transplantation within Singapore**

Deignan, Lindsey K. and McDougald, Diane


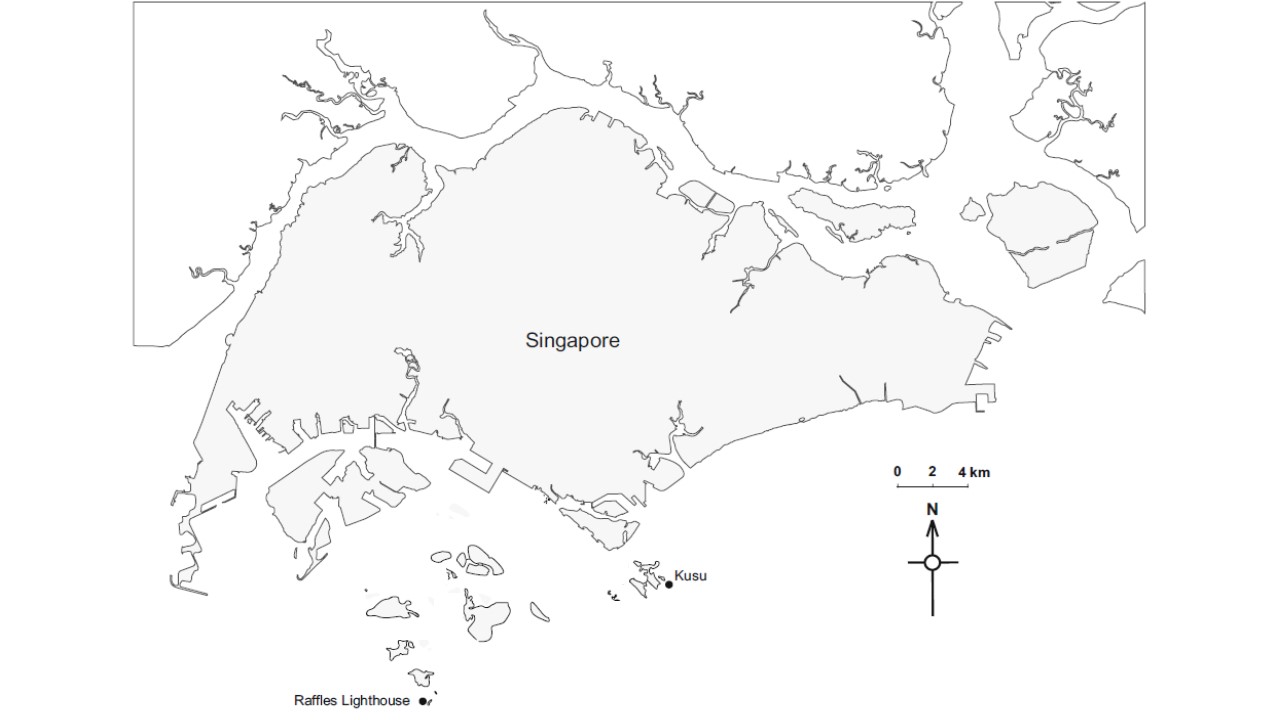


**Fig. S1**  Map of transplantation sites adapted from Wainwright et al. (2019) showing Raffles Lighthouse and Kusu Island.


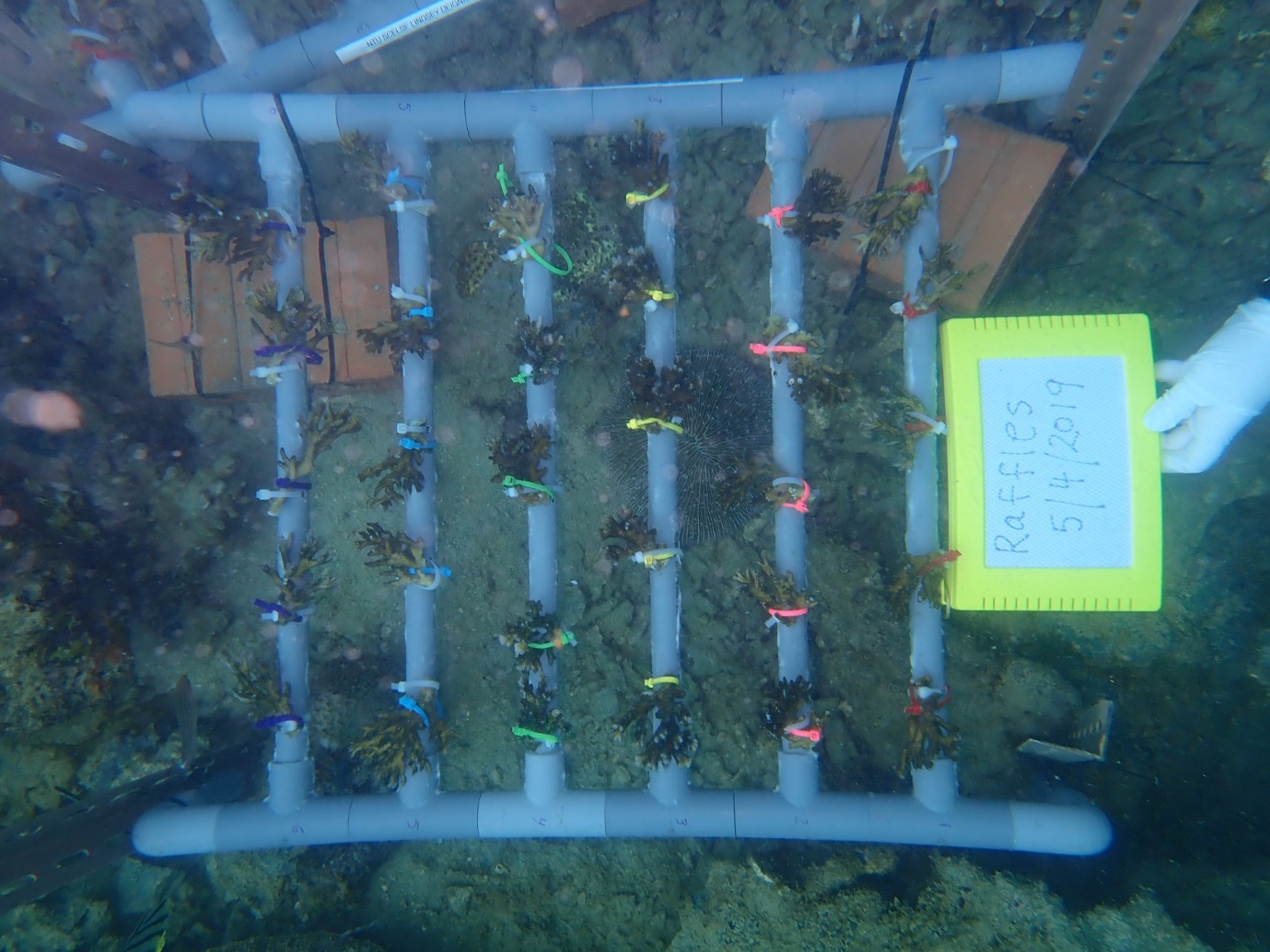


**Fig. S2** Example of the PVC frame with attached coral fragments from Raffles.


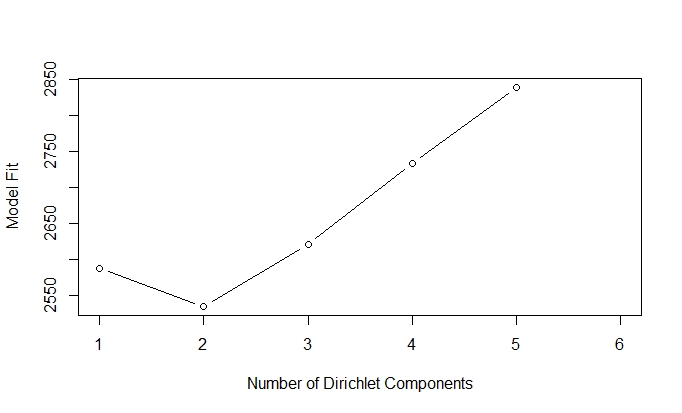


**Fig. S3** DMM model results showing two Dirichlet mixture component of the coral samples, based on the Laplace model goodness-of-fit test before the removal of four contaminating AVS.


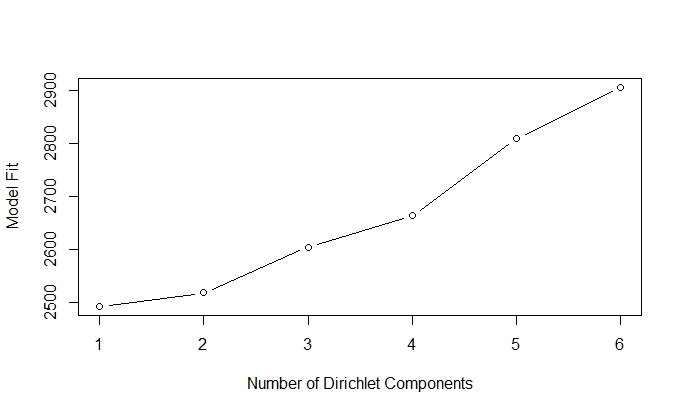


**Fig. S4** DMM model results showing one Dirichlet mixture component of the coral samples, based on the Laplace model goodness-of-fit test following the removal of the following four contaminating AVS:

| Phylum | Genus | Sequence |  |
| --- | --- | --- | --- |
| Proteobacteria | *Ralstonia* | CCGCGGTAATACGTAGGGTCCAAGCGTTAATCGGAATTACTGGGCGTAAAGCGTGCGCAGGCGGTTGTGCAAGACCGATGTGAAATCCCCGGGCTTAACCTGGGAATTGCATTGGTGACTGCACGGCTAGAGTGTGTCAGAGGGGGGTAGAATTCCACGTGTAGCAGTGAAATGCGTAGAGATGTGGAGGAATACCGATGGCGAAGGCAGCCCCCTGGGATAACACTGACGCTCATGCACGAAAGCGTGGGGAGCAAACAGGATTAGAAACC |  |
| Proteobacteria | *Pseudomonas* | CCGCGGTAATACGAAGGGTGCAAGCGTTAATCGGAATTACTGGGCGTAAAGCGCGCGTAGGTGGTTCAGCAAGTTGGATGTGAAATCCCCGGGCTCAACCTGGGAACTGCATCCAAAACTACTGAGCTAGAGTACGGTAGAGGGTGGTGGAATTTCCTGTGTAGCGGTGAAATGCGTAGATATAGGAAGGAACACCAGTGGCGAAGGCGACCACCTGGACTGATACTGACACTGAGGTGCGAAAGCGTGGGGAGCAAACAGGATTAGATACC |  |
| Proteobacteria | *Ralstonia* | CCGCGGTAATACGTAGGGTCCAAGCGTTAATCGGAATTACTGGGCGTAAAGCGTGCGCAGGCGGTTGTGCAAGACCGATGTGAAATCCCCGGGCTTAACCTGGGAATTGCATTGGTGACTGCACGGCTAGAGTGTGTCAGAGGGGGGTAGAATTCCACGTGTAGCAGTGAAATGCGTAGAGATGTGGAGGAATACCGATGGCGAAGGCAGCCCCCTGGGATAACACTGACGCTCATGCACGAAAGCGTGGGGAGCAAACAGGATTAGATACC |  |
| Proteobacteria | *Pseudomonas* | CCGCGGTAATACGAAGGGTGCAAGCGTTAATCGGAATTACTGGGCGTAAAGCGCGCGTAGGTGGTTCAGCAAGTTGGATGTGAAATCCCCGGGCTCAACCTGGGAACTGCATCCAAAACTACTGAGCTAGAGTACGGTAGAGGGTGGTGGAATTTCCTGTGTAGCGGTGAAATGCGTAGATATAGGAAGGAACACCAGTGGCGAAGGCGACCACCTGGACTGATACTGACACTGAGGTGCGAAAGCGTGGGGAGCAAACAGGATTAGAAACC |  |


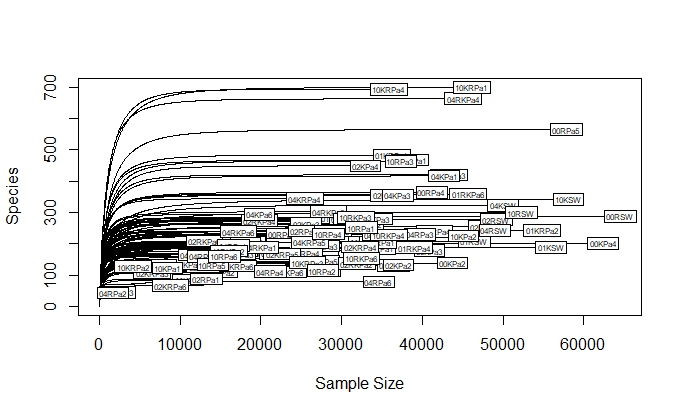


**Fig. S5** Rarefaction curve of all samples, including those samples which were excluded for failing to reach a asymptotic levels.


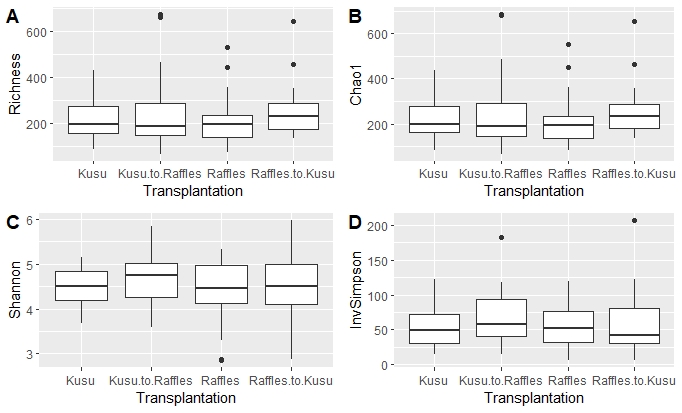


**Fig S6** Alpha diveristy metrics (richness, Chao1, Shannon diversity, and inverse Simpson) ere not significantly among the resident and transplant groups.


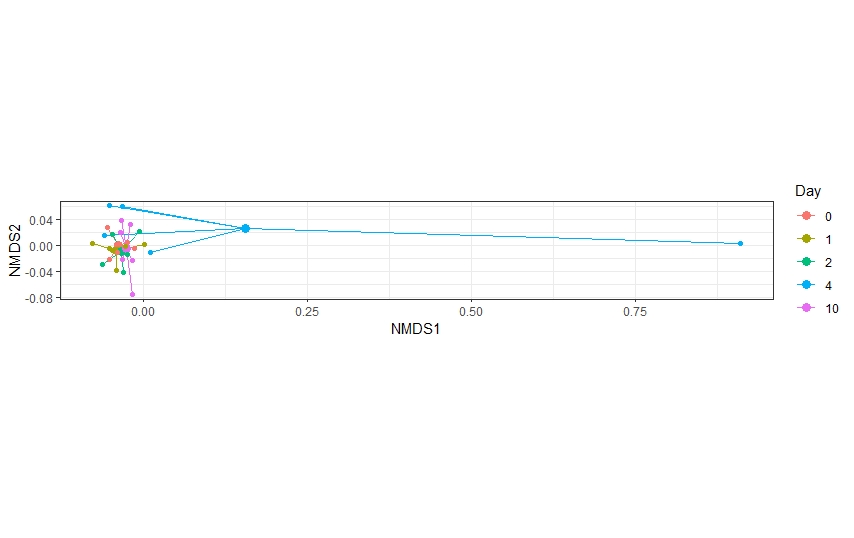


**Fig. S7**  nMDS plot of the Resident Raffles samples at each sampling day graphically represented as a spider plot connecting each sample with the daily group centroid.

**Table S1** The number of coral colonies analyzed for each sample group at the five time points.

|  | Resident Raffles | Resident Kusu | Transplant RK | Transplant KR |
| --- | --- | --- | --- | --- |
| Day 0 | 6 | 6 | - | **-** |
| Day 1 | 6 | 6 | 5 | 6 |
| Day 2 | 5 | 5 | 6 | 5 |
| Day 4 | 5 | 5 | 4 | 5 |
| Day 10 | 6 | 3 | 5 | 3 |

**Table S2** Main PERMANOVA test results (subsequent pairwise comparisons for each main test are reported in Table 2)

| **Two-factor PERMANOVA table of results using Location and Time as factors** | | | | | |
| --- | --- | --- | --- | --- | --- |
| Source | df | SS | MS | Pseudo-F | P(perm) |
| Lo | 1 | 6623.6 | 6623.6 | 2.013 | 0.001 |
| Ti | 3 | 15534 | 5178.1 | 1.5737 | 0.001 |
| LoxTi | 3 | 12469 | 4156.5 | 1.2632 | 0.001 |
| Res | 72 | 2.37E+05 | 3290.4 |  |  |
| Total | 79 | 2.71E+05 |  |  |  |
|  |  |  |  |  |  |
| **Two-factor PERMANOVA table of results using Origin and Time as factors** | | | | | |
| Source | df | SS | MS | Pseudo-F | P(perm) |
| Or | 1 | 6139 | 6139 | 1.8495 | 0.001 |
| Ti | 3 | 15390 | 5130.1 | 1.5456 | 0.001 |
| OrxTi | 3 | 10589 | 3529.5 | 1.0634 | 0.17 |
| Res | 72 | 2.39E+05 | 3319.2 |  |  |
| Total | 79 | 2.71E+05 |  |  |  |
|  |  |  |  |  |  |
| **Two-factor PERMANOVA table of results using Origin and Time as factors within Raffles** | | | | | |
| Source | df | SS | MS | Pseudo-F | P(perm) |
| Or | 1 | 5623.4 | 5623.4 | 1.6327 | 0.001 |
| Ti | 3 | 15584 | 5194.6 | 1.5082 | 0.001 |
| OrxTi | 3 | 10802 | 3600.8 | 1.0455 | 0.255 |
| Res | 33 | 1.14E+05 | 3444.2 |  |  |
| Total | 40 | 1.45E+05 |  |  |  |
|  |  |  |  |  |  |
| **Two-factor PERMANOVA table of results using Origin and Time as factors within Kusu** | | | | | |
| Source | df | SS | MS | Pseudo-F | P(perm) |
| Or | 1 | 4318.4 | 4318.4 | 1.4533 | 0.001 |
| Ti | 3 | 12427 | 4142.3 | 1.3941 | 0.001 |
| OrxTi | 3 | 10326 | 3442 | 1.1584 | 0.011 |
| Res | 31 | 92112 | 2971.4 |  |  |
| Total | 38 | 1.19E+05 |  |  |  |

**Table S3** Pairwise PERMDISP analysis among sample groups.

|  | t | P |
| --- | --- | --- |
| Resident Raffles, Resident Kusu | 2.1854 | 0.046 |
| Resident Raffles, Transplant RK | 3.7042 | **0.002** |
| Resident Raffles, Transplant KR | 0.1496 | 0.898 |
| Resident Kusu, Transplant KR | 2.2899 | 0.03 |
| Resident Kusu, Transplant RK | 1.5003 | 0.187 |
| Transplant KR, Transplant RK | 3.9894 | **0.002** |

**Table S3** Seawater nutrient concentrations at Raffles and Kusu during the experimental period, and the test results indicating any significant differences in nutrient concentration between reefs.

| Raffles seawater nutrient concentrations at each time point, including the mean (±standard deviation). All concentrations are µmol/L. | | | | | | |
| --- | --- | --- | --- | --- | --- | --- |
|  | NOx | Nitrate | Nirite | Ammonia | Phosphate | Silicate |
| Day 0 | 0.755 | 0.755 | ND | 0.191 | 0.061 | 4.416 |
| Day 1 | 0.775 | 0.775 | ND | 0.26 | 0.063 | 4.344 |
| Day 2 | 0.893 | 0.893 | ND | 0.247 | 0.055 | 3.664 |
| Day 4 | 0.908 | 0.958 | ND | 0.056 | 0.052 | 3.088 |
| Day 10 | 1.272 | 1.25 | 0.027 | 0.131 | 0.078 | 3.639 |
| Mean | 0.921±0.208 | 0.926±0.199 | 0.027 | 0.177±0.085 | 0.062±0.010 | 3.830±0.553 |
|  |  |  |  |  |  |  |
| Kusu seawater nutrient concentrations at each time point, including the mean (±standard deviation). All concentrations are µmol/L. | | | | | | |
|  | NOx | Nitrate | Nirite | Ammonia | Phosphate | Silicate |
| Day 0 | 0.479 | 0.479 | ND | 0.099 | 0.049 | 4.365 |
| Day 1 | 0.397 | 0.397 | ND | 0.16 | 0.032 | 3.607 |
| Day 2 | 0.503 | 0.503 | ND | 0.143 | 0.063 | 3.547 |
| Day 4 | 0.842 | 0.803 | 0.04 | 0.157 | 0.102 | 3.945 |
| Day 10 | 0.666 | 0.608 | 0.059 | ND | 0.058 | 3.783 |
| Mean | 0.577±0.177 | 0.558±0.156 | 0.049±0.013 | 0.140±0.028 | 0.061±0.026 | 3.849±0.328 |
|  |  |  |  |  |  |  |
| T-test comparing seawater nutrients between Raffles and Kusu. | | | | | |  |
|  | t | df | p-value |  |  |  |
| Nox | 2.8085 | 7.8034 | 0.02347 |  |  |  |
| Nitrate | 3.2491 | 7.5673 | 0.01264 |  |  |  |
| Nitrite | NA | NA | NA |  |  |  |
| Ammonia | 1.3486 | 7.5989 | 0.2163 |  |  |  |
| Phosphate | 0.080509 | 5.1875 | 0.9388 |  |  |  |
| Silicate | -0.06681 | 6.5028 | 0.9487 |  |  |  |
